# Comparison of Tooth Color and Enamel and Dentinal Thickness between Orthodontically Treated and Untreated Individuals

**DOI:** 10.3390/diagnostics13122066

**Published:** 2023-06-14

**Authors:** Zaki Hakami, Hussain YA Marghalani, Ismaeel Hedad, Mazen Khawaji, Ghadah Abutaleb, Amjad Hakami, Salem Almoammar, Abdulrahman Alshehri

**Affiliations:** 1 Division of Orthodontics, Department of Preventive Dental Sciences, College of Dentistry, Jazan University, Jazan 45142, Saudi Arabia; aalshehri@jazanu.edu.sa; 2 Orthodontic Department, Faculty of Dentistry, King Abdulaziz University, Jeddah 21589, Saudi Arabia; hymarghalani@kau.edu.sa; 3 Jazan Dental Specialists Center, Ministry of Health, Jazan 82943, Saudi Arabia; somaah22@gmail.com; 4 Prince Fahd Bin Sultan Primary Care, Ministry of Health, Tabuk 47311, Saudi Arabia; mazen.khawaji@gmail.com; 5 Samtah General Hospital, Ministry of Health, Jazan 23437, Saudi Arabia; dr.ghadahkhalid@gmail.com; 6 Private Dental Center, Jazan 84521, Saudi Arabia; hakamiamjad2@gmail.com; 7 Department of Orthodontics and Pediatric Dentistry, College of Dentistry, King Khalid University, Abha 61421, Saudi Arabia; smalmoamr@kku.edu.sa

**Keywords:** cone-beam computed tomography, tooth discoloration, enamel, dentin, color, orthodontic, tooth movement

## Abstract

This study compared tooth color and enamel and dentinal thickness between orthodontically treated and untreated individuals. (1) Methods: A matched case–control study was conducted. The sample included 26 patients who had received orthodontic treatment and 31 matched controls. The color assessment was performed subjectively using the VITA 3D-Master (VM) shade guide and objectively using the VITA Easyshade (VE) spectrophotometer. Differences in L*, a*, and b* (lightness, red/green, and blue/yellow) were calculated. The color change was evaluated using ΔE*ab and the whiteness index (*WID*). Tooth structure thickness (labiolingual, labial enamel, and labial dentin) was evaluated using cone-beam computerized tomography. The correlations between overall tooth color and tooth structure thickness were evaluated. (2) Results: A total of 228 teeth were evaluated. Color assessment using VM showed significant differences between orthodontically treated and untreated teeth (*p* < 0.001), while VE revealed no significant differences. Both groups showed no difference in tooth enamel and dentinal thickness. Significant differences in tooth color (*p* < 0.05) were observed between genders. Both VM and VE showed weak to moderate correlations with tooth color and enamel and dentinal thickness (*p* < 0.05). (3) Conclusions: Orthodontic treatment may demonstrate visually perceptible but acceptable and clinically undetectable tooth color alteration.

## 1. Introduction

Orthodontic treatment is performed to attain occlusal harmony and meet esthetic demands [1]. Nevertheless, many adverse effects on tooth structure have been reported following orthodontic treatment. These effects include white spot formation, decalcification, microcracks, abrasions, and discoloration caused by pulp necrosis or devitalization [2,3]. Several preventive strategies have been employed, ranging from the selection of the adhesive system for bonding orthodontic brackets to techniques for and after debonding and removal of adhesive remnants [4,5,6].

Tooth color, which is influenced by light and the enamel surface, can be mainly assessed by two means: visual and instrumental assessment [7]. Visual color assessment with color tabs, which is still the most popular method, is highly subjective. General factors that can lead to inconsistencies in human perception of color include age, fatigue, emotions, examiner’s experience, light source, room conditions, and physiological variables such as color-deficient vision [8,9]. The transformation of the Commission Internationale de l’Eclairage system into numeric data and advances in computer and optical technologies have made tooth color evaluation an objective method [10]. Several studies have concluded that the instrumental color assessment method could provide a precise and reliable measurement of tooth color in humans that is more accurate and reproducible compared with the visual method [11,12,13,14].

Many in vitro studies have shown enamel color changes resulting from bracket placement, removal, and cleaning procedures [15,16,17,18]. Light scattering of the area underneath the brackets is influenced by many factors, including acid etching, penetration of resin tags into the enamel surface, removal of residual resin, and increased roughness [4,15,19,20]. It has been found that tooth color alteration occurs despite the method used for enamel surface preparation for bonding or removal of the adhesive material [4]. Moreover, another study found that both the burs used for the removal of adhesive material and the adhesive material itself had an impact on tooth color change [19]. These findings have been confirmed in several clinical trials, where obvious enamel color alterations may occur after orthodontic treatment with fixed appliances [21].

Changes in enamel thickness following orthodontic treatment in relation to the proximal site, gender, and certain ethnicities have been reported in previous studies [22]. However, these factors do not fully explain the exact reason behind differences in enamel thickness. Ultrasound [23], cone-beam computed tomography (CBCT) [24], optical coherence tomography [25,26], and quantitative light-induced fluorescence technology [27] are the various techniques that have been used to evaluate enamel and dentin thickness.

In addition, orthodontic treatment induces changes in the pulp–dentin complex, causing a deposition of tertiary dentin, which leads to a reduction in the pulp volume [28,29]. It is well known that tooth discoloration can result from alteration of the dentin thickness [30,31]. Various studies have evaluated dentin thickness after endodontic and prosthodontic procedures using CBCT [24,32]. To the best of our knowledge, no study has documented the correlation between tooth color changes with enamel and dentin thickness in subjects who had undergone orthodontic treatment and who had not. Hence, this study was undertaken to compare tooth color and structure thickness between orthodontically treated and untreated individuals and between genders.

## 2. Materials and Methods

A case–control study was conducted among the dental students of Jazan University. The study was approved by the Scientific Research Ethics Committee (approval reference number: REC41/1-019), and subjects read and signed an informed consent form.

### 2.1. Subjects and Teeth

The treatment group was students who had orthodontic treatment completed at least six months prior to study enrollment. Controls were students who had not undergone orthodontic treatment in their lifetime. Cases and controls were matched by age, race, marital status, and level of education. A questionnaire (Google Form) was sent to 394 dental students who were currently studying at the College of Dentistry. Around 222 students responded and were willing to participate in the study. Among these students, 89 individuals (48 males and 41 females) had received CBCT.

Sample size calculation was performed based on a previous study [33], in which the mean lightness value for the control group was considered as 73.43 ± 2.4. For the cases, the corresponding value was proposed as 71.42 ± 2.4. The power was set at 80% and the significance value at 0.05. The calculation yielded a sample size of 48 subjects (24 for each group).

Clinical examination was implemented with the following inclusion criteria: (1) no missing or impacted maxillary anterior teeth; (2) no pathological lesions related to teeth; (3) no carious lesions or restorations; (4) no enamel decalcifications; (5) no severe plaque accumulation; (6) no history of bleaching; (7) no history of daily use of chlorohexidine mouth rinses; (8) medically and mentally fit with no disabilities; and (9) availability of a CBCT image showing at least the anterior maxilla. The teeth examined were maxillary centrals (#11), laterals (#22), canines (#13), and first premolars (#24). After applying exclusion and inclusion criteria, a total of 57 students were recruited, with 26 students in the case group and 31 students in the control group.

### 2.2. Color Measurements

The VITA Toothguide 3D-Master (VM) system (VITA Zahnfabrik GmbH, Bad Säckingen, Germany) was used as a visual technique to measure the middle region of the labial surface of the tooth. Before shade selection, the teeth were cleaned using polishing brushes and paste to remove any accumulated plaque and stains. Because the shade of a tooth becomes lighter when dry, the measurement was performed within 30 s of moistening. According to the manufacturer’s instructions, the examiner determined the lightness from black to white first, the chroma from pale to strong second, and the hue from yellow or red third [34]. To minimize human factors affecting tooth shade selection, two trained dentists performed the measurements separately from each other. All patients were examined in the morning session at the same clinic using good lighting conditions. During shade selection, the lamps in the units were switched off, and the patients were in an upright position. None of the examiners had a positive history of visual color deficiency.

The VITA Easyshade (VE; VITA Zahnfabrik GmbH) device provides precise color measurements. The color assessment was based on three color parameters: lightness (L), red/green chromaticity (a), and yellow/blue chromaticity (b). The device was calibrated according to the manufacturer’s instructions, and an infection control shield was placed on the probe tip before each measurement. The operating mode of the device was selected for the measurement of a single tooth. The device tip was held at a right angle to the middle region of the labial surface of the tooth. The measuring tip was held unmoving until the instrument beeped. Three consecutive measures for each color parameter were performed for the teeth, and the average value was recorded. The color values assessed were lightness, chroma, H degree, red/green chromaticity, yellow/blue chromaticity [35], and the whiteness index (*WID*) [36]. The color values of each shade tab were obtained according to Ahn and Lee [37].

The CIELab color difference (ΔE*ab) was calculated as follows [35]:

ΔE*ab = [(ΔL*)^2^ + (Δa*)^2^ + (Δb*)^2^] ½ (1)

where ΔL*, Δa*, and Δb* correspond to the difference in lightness, red/green chromaticity, and yellow/blue chromaticity, respectively, between the control and treatment groups, which were calculated separately for VM and VE. The perceptibility threshold was ΔE*ab = 1.2, and the acceptability threshold was ΔE*ab = 2.7. For the whiteness index (*WID*), the following equation was used [36]:

*WID* = 0.511L* − 2.324a* − 1.100b* (2)

The difference in the *WID* between the control and treatment groups (Δ*WID*) was calculated for VM and VE separately.

### 2.3. CBCT Measurements

**Figure 1:**
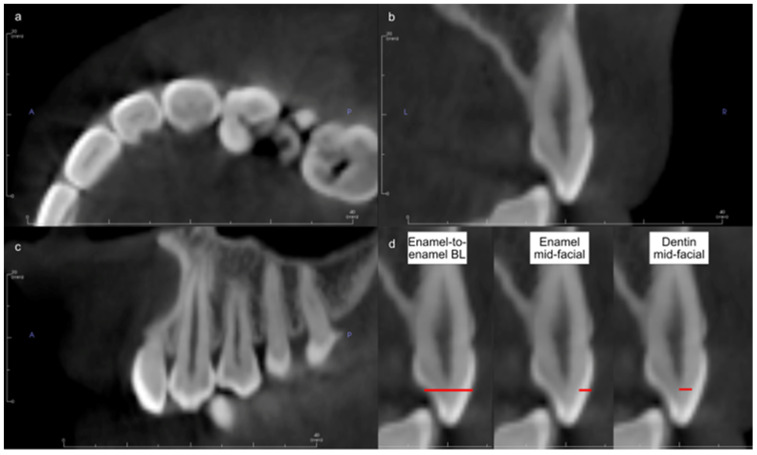
Example of the orientation of the CBCT image for the canine in (**a**) occlusal, (**b**) proximal, and (**c**) facial views. (**d**) Red lines used to measure tooth structure thicknesses.

Acquisition of CBCTs was accomplished using the 3D Accuitomo 170 CBCT Machine (J Morita Mfg. Corp., Kyoto, Japan) at 90 kVp and 5–8 mA. CBCTs were imported into Invivo software (version 6.5; Osteoid Inc., Santa Clara, CA, USA). To locate the cross-sectional axis of each tooth, a line was drawn in the middle of the tooth mesiodistally from the occlusal view. From the proximal view, the cross-section of the tooth was adjusted to show the largest buccolingual width for the pulp of the selected tooth. A buccolingual line was drawn, connecting the middle of the occlusogingival surfaces of the labial and lingual sides. This line was used to measure the enamel and dentin thicknesses in mm (Figure 1). The measurements were performed blindly by a single examiner.

### 2.4. Data Management and Analysis

Data were analyzed using SPSS version 27 (IBM corporation, Armonk, NY, USA). Based on the Shapiro–Wilk test, the normality assumption was rejected (*p* < 0.05). Therefore, nonparametric tests were used. The intraclass correlation coefficient yielded moderate reliability (ICC = 0.525 for single measures, ICC = 0.688 for average measures). Descriptive variables were expressed in terms of medians and interquartile ranges. The Mann–Whitney U-test was used to evaluate the tooth color difference and tooth structure thickness across gender and differences in tooth color and tooth structure thickness between the treatment and control groups. Spearman’s correlation was used to find the relationship between tooth color and enamel and dentinal thickness.

## 3. Results

A total of 228 teeth were evaluated. Table 1 and Table 2 show the overall teeth structure thicknesses and color values, respectively, for the control and case groups. A significant difference in tooth color between the orthodontically treated and untreated individuals was observed when color values were measured subjectively using VM. However, when objective measurements using VE were used, there was no significant difference.

Analysis of the color variables obtained via VM revealed statistically significantly higher values for chroma, a*, and b* in the case group compared to the control group (*p* < 0.001). For the L* value, there was no significant difference, but the H degree was significantly lower in the case group compared to the control group (*p* < 0.001) (Table 3).

According to CIELAB-based *WID*, higher values indicate more whiteness and vice versa. The results showed significantly higher values of *WID* for people who had not undergone orthodontic treatment compared to those who had treatment when the measurements were obtained by VM (*p* < 0.001) (Table 3). According to Paravina et al. [38], Δ*E* demonstrated visually perceptible but acceptable color alteration (Δ*E* = 1.28). Moreover, it was clinically undetectable according to Karamouzos et al.’s [13] standard value of clinical detection (Δ*E* = 3.7).

Regarding the distribution of color values between gender, a significant subjective and objective difference in tooth color between males and females was observed using the VM and VE methods, respectively (Table 4).

Regarding the comparison of tooth structure thickness between treated and untreated subjects, no significant difference was observed for the buccolingual thickness, labial enamel thickness, or labial dentine thickness (Table 5). Moreover, no significant differences between genders were observed for any of the measured tooth structure thicknesses (Table 6).

Table 7 shows the correlation between tooth color and thickness. Both VM and VE showed significant weak to moderate correlations between tooth color and structure thickness. Relatively higher correlations were observed for measurements obtained by VE compared to VM. The values of chroma, a*, and b* showed significantly positive correlations with labiolingual tooth thickness, labial enamel thickness, and labial dentin thickness. However, tooth structure thickness showed significantly negative correlations with L*, H* degree, and *WID* values.

## 4. Discussion

Orthodontic treatment, which creates biological challenges for the stomatognathic system, is used to obtain perfection in occlusal functions and, more recently, to achieve tooth esthetics. Tooth color is a major factor in dental esthetics [39].

Many factors in the oral cavity influence natural tooth color, including ambient lighting conditions, light dispersed from nearby perioral and gingival tissues, and resting salivary flow rates, which alter tooth hydration and, eventually, the reflective index of the underlying surface. Visual determination and instrumental measurement are the two most popular approaches for measuring perceived tooth color. Despite the fact that visual determination is subjective, it is still the most commonly used method of color determination in dentistry. Due to the increasing desire for objective color matching in dentistry and rapid advancements in optical electronic sensors and computer technology, instrumental measuring equipment has become a supplement to the visual tooth color evaluation [33]. During shade matching, the value should be decided first, followed by chroma. Hue is decided last by comparing it to the value and chroma shade tabs. Most shade guides are prone to coverage problems, which occur when the shade range in the guide does not match the color range of natural teeth. External light conditions, experience, age, and human eye fatigue are the inherent limitations of current shade guides [37].

Tooth color esthetics play a vital role in the visual evaluation of a completed orthodontic case. Clinical performance and patient satisfaction are affected by changes in the color of the enamel surface. Numerous studies have evaluated enamel color change following orthodontic treatment using bonding methods and clean-up, whereas changes in tooth structure thickness following orthodontic treatment are less evaluated [40,41,42,43,44]. An in vitro study conducted by Seeliger et al. [26] did not find statistically significant differences in average enamel thickness among orthodontically treated and untreated groups using two-dimensional optical coherence tomography. Hence, this study was performed to compare tooth color and structure thickness between orthodontically treated and untreated individuals using a case–control study design. In our research, statistically significant differences were not observed for tooth color among orthodontically treated and untreated groups using VE. Similarly, the studies conducted by Wriedt et al. [45] and Trakyali et al. [18] found that bonding and debonding methods had no discernible effect on the color of bovine or human enamel. In another study conducted by Karamouzos et al. [33], L* values decreased, but a* and b* values increased when tooth color was measured before and after orthodontic treatment, with tooth color darkening and moving toward more red and yellow color ranges. This finding is in line with our finding that a* and b* values increased according to the subjective assessment using VM.

Post-debonding resin removal methods, which involve grinding with various devices and the penetration of resin tags into the enamel structure, could be linked to changes in enamel color after orthodontic treatment [21]. Resin impregnation in the enamel structure cannot be reversed by debonding and cleaning procedures, and, hence, enamel color changes may occur as a result of the direct absorption of food colorants and products resulting from orthodontic appliance corrosion due to a change in the refractive index of the region, or by altering the diffusely reflected light component [15,16,17]. Moreover, altered enamel morphology and texture lost during debonding cannot be restored with polishing media during the post-finishing stages. These irreversible changes could possibly affect the optical properties of the enamel surface, such as gloss and luster, subsequently influencing the color parameters of natural teeth, which is different from the natural aging of teeth [18]. The application of remineralizing solutions such as biomimetic nano-hydroxyapatite before bonding orthodontic appliances could be effective in reducing the incidence of enamel demineralization and discoloration [46,47].

Orthodontic treatment creates alterations in the blood flow and leads to the release of inflammatory mediators that stimulate odontoblasts, causing tertiary dentin deposition, reducing pulpal space, and increasing dentin thickness. Venkatesh et al. [29] found a significant reduction in pulp size in all anterior teeth of 48 patients after orthodontic treatment. In our study, the dentin thickness was slightly greater, but not significantly, in the orthodontically treated individuals than the untreated ones. Thus, the reduced pulp volume after orthodontic treatment that was reported by Venkatesh et al. [29] could have been due to tertiary dentin deposition in the root. Nevertheless, future studies with larger sample sizes may reveal further information.

Vital teeth tend to be more reddish, particularly in the cervical region, than extracted teeth owing to the role of the gingiva [48]. Our in vivo study showed a weak to moderate correlation between labial enamel, dentin, and color values. These results are in agreement with the study of He et al. [49], who found that color differences are weakly to moderately correlated with variations in the enamel thickness. Tooth color tone has been suggested to be predominately determined by dentin properties, whereas enamel plays a major role in refraction and the scattering of light [50]. In the present study, tooth color was more correlated with labial dentine thickness and labiolingual tooth thickness compared to labial enamel thickness. This supports the finding that the color of a complete tooth correlated strongly to that of a tooth with a trimmed labial enamel [50].

Several studies have evaluated visual thresholds of natural teeth for color perceptibility and acceptability in dentistry [38]. A CIELab (ΔE*ab) of 3.7 units has been proposed as the clinical acceptance limit for tooth color matching [33]. Karamouzos et al. [33] reported tooth color alterations associated with fixed orthodontic treatment, with 80% of patients having at least one tooth with an unacceptable color change. Moreover, a longitudinal evaluation of tooth color after orthodontic treatment revealed visible but clinically acceptable changes [20]. In our study, ΔE*ab was calculated from the mean difference between the control and treatment groups. Δ*E* for VM and VE color assessment methods were 1.28 and 0.77, respectively, which were not clinically perceptible.

Complementary to color assessment, Paravina et al. [38] recommended that the perceptibility and acceptability thresholds for CIELab (ΔE*ab) among observers of different backgrounds should be 1.2 and 2.7, respectively. In this study, the value of Δ*E* was 1.28, which is above the perceptibility threshold but below the acceptability threshold for color measurements performed by VM. Nevertheless, when color measurements were performed by VE, Δ*E* was 0.77, which is below both the perceptibility and acceptability thresholds. Thus, according to previously established threshold values for color difference perception, people who had received orthodontic treatment might experience perceptible, but still acceptable, tooth color alteration.

Recently, *WID* has been widely used in dentistry for the clinical assessment of whitening treatments. It is customized, easy to use, and strongly correlated with the average visual perception [36]. The thresholds of *WID* for perceptibility and acceptability of color differences have been investigated for dentistry and dental applications. Perez et al. [51] found that the 50:50% perceptibility thresholds and 50:50% acceptability thresholds for *WID* were 0.72 and 2.60, respectively. Moreover, these threshold values were different between dentists and laypersons, as dentists showed lower threshold values and better discrimination ability [51]. In the present study, Δ*WID* of VM and VE color assessment methods were 2.26 and 0.28, respectively. A subjective assessment using VE might show only perceptible whiteness variation between orthodontically treated and untreated individuals. 

Regarding gender differences, both methods of tooth color values showed significant differences between males and females. This was in accordance with the study conducted by Esan et al. [52], in which men presented with darker tooth shades than women in the same age group. Moreover, in our study, no gender differences were observed with respect to the labiolingual thickness, labial enamel, and labial dentin thickness. A recently published systematic review about proximal enamel thickness reported no differences between genders [53].

This case–control study evaluated differences in tooth color and structure thickness among orthodontically treated and untreated individuals. In order to reduce heterogeneity, all study subjects that were included belonged to a defined population with related background characteristics and similar knowledge about oral hygiene. This can be considered a strength of this research. Nevertheless, random errors during the quantitative evaluation of tooth color and the precision of CBCT measurements were the major limitations of our study. These random errors were minimized by repeated measurements and averaging and also by acquiring better control of methodological and environmental factors. Moreover, variations of the human eye for discriminating colors are confounding limitations. However, previous studies showed that the discrimination threshold reduced with experience and training [54]. In our study, the tooth color was measured by experienced observers.

## 5. Conclusions

This study compared tooth color and structure thickness between people who had received orthodontic treatment and those who had not received any treatment. Subjective measurements using shade tabs revealed significant differences with higher values for chroma, a*, and b* and lower values for H degrees and *WID* in the case group, while objective instrumental measurements showed no significant differences. Males exhibited darker tooth shades compared to females of the same age group, while no difference in tooth structure thickness was reported. Moreover, a significant but weak to moderate correlation was observed between tooth color and structure thickness. From this research, it can be concluded that orthodontic treatment may demonstrate visually perceptible, but acceptable, and clinically undetectable tooth color alteration without major effects on tooth structure thickness. Future larger-scale clinical trials will be necessary to further expand the available knowledge concerning the relationship between tooth color and tooth structure thickness after orthodontic treatment.

## Figures and Tables

**Table 1:** Descriptive statistics of tooth structure thickness.

Thickness	Overall	Control	Case
	Mean ± SD	Mean ± SD	Mean ± SD
Enamel-to-enamel buccolingual	5.97 ± 1.91	5.89 ± 1.89	6.08 ± 1.94
Enamel mid-facial	1.24 ± 0.19	1.24 ± 0.19	1.24 ± 0.22
Dentin mid-facial	1.87 ± 0.84	1.85 ± 0.84	1.89 ± 0.84

**Table 2:** Descriptive statistics of color variables.

Measurement Method	Color Value	Overall (*n* = 228) Mean ± SD	Control (*n* = 124) Mean ± SD	Case (*n* = 104) Mean ± SD
Age		23.91	24.29	23.46
Vita 3D-Master	L*	56.86 ± 1.99	57.13 ± 2.03	56.54 ± 1.91
	Chroma	11.35 ± 2.69	10.83 ± 2.58	11.97 ± 2.69
	H degree	80.94 ± 2.22	81.31 ± 2.38	80.5 ± 1.93
	a*	1.84 ± 0.69	1.69 ± 0.70	2.01 ± 0.65
	b*	11.2 ± 2.63	10.71 ± 2.53	11.8 ± 2.64
	*WID*	14.74 ± 4.74	15.77 ± 4.73	13.51 ± 4.47
Vita Easy Shade	L*	59.6 ± 2.85	59.36 ± 2.98	59.89 ± 2.66
	Chroma	11.25 ± 2.67	10.99 ± 2.69	11.55 ± 2.62
	H degree	83.6 ± 2.86	83.45 ± 3.03	83.79 ± 2.66
	a*	1.29 ± 0.69	1.31 ± 0.73	1.29 ± 0.66
	b*	11.15 ± 2.62	10.89 ± 2.64	11.45 ± 2.58
	*WID*	17.56 ± 4.90	17.69 ± 5.04	17.41 ± 4.75

**Table 3:** Comparison of the median and interquartile range (IQR) tooth color values between the control and case groups using Vita 3D-Master and Vita Easy Shade.

	Color Value	Control Median (IQR)	Case Median (IQR)	*p* Value *
Vita 3D-Master	L*	56.6 (1.8)	56.6 (3.4)	0.066
	Chroma	11.0 (2.9)	11.6 (3.5)	0.001 *
	H degree	81.9 (3.6)	80.2 (2.5)	0.002 *
	a*	1.6 (0.8)	2.0 (0.9)	0.001 *
	b*	10.9 (2.7)	11.4 (3.4)	0.001 *
	*WID*	16.3 (5.5)	12.5 (6.6)	0.001 *
Vita Easy Shade	L*	60.6 (5.1)	61.3 (3.6)	0.179
	Chroma	10.5 (3.4)	11.0 (3.4)	0.131
	H degree	83.5 (3.6)	83.6 (3.6)	0.511
	a*	1.1 (0.8)	1.1 (0.8)	0.989
	b*	10.5 (3.4)	10.9 (3.5)	0.115
	*WID*	16.5 (5.4)	16.5 (5.4)	0.635

* Mann–Whitney U-test is significant at *p* < 0.05.

**Table 4:** Comparison of median and interquartile range (IQR) tooth color values between males and females using Vita 3D-Master and Vita Easy Shade.

	Color Value	Male (*n* = 120) Median (IQR)	Female (*n* = 108) Median (IQR)	*p* Value *
Vita 3D-Master	L*	57.1 (2.7)	56.4 (3.4)	0.012 *
	Chroma	11.0 (4.2)	11.0 (3)	0.001 *
	H degree	81.9 (2.5)	81.1 (2.5)	0.257
	a*	1.6 (0.9)	1.6 (0.8)	0.030 *
	b*	10.9 (3.9)	10.9 (2.9)	0.001 *
	*WID*	16.30 (6.78)	16.30 (5.45)	0.001 *
Vita Easy Shade	L*	61.3 (3.6)	60.6 (5.1)	0.399
	Chroma	11.0 (4.2)	10.5 (1.8)	0.008 *
	H degree	83.6 (3.6)	83.5 (3.6)	0.859
	a*	1.1 (0.8)	1.1 (0.8)	0.481
	b*	10.9 (4.2)	10.5 (2)	0.009 *
	*WID*	16.30 (5.37)	17.07 (5.37)	0.128

* Mann–Whitney U-test is significant at *p* < 0.05.

**Table 5:** Comparison of the median and interquartile range (IQR) tooth structure thicknesses between the control and case groups.

	Control Median (IQR)	Treatment Median (IQR)	*p* Value *
Enamel-to-enamel buccolingual	5.37 (2.79)	5.50 (3.52)	0.507
Enamel mid-facial	1.22 (0.25)	1.21 (0.27)	0.803
Dentin mid-facial	1.53 (1.02)	1.55 (1.48)	0.656

* Mann–Whitney U-test is not significant.

**Table 6:** Comparison of the median and interquartile range (IQR) tooth structure thicknesses between males and females.

	Male Median (IQR)	Female Median (IQR)	*p* Value *
Enamel-to-enamel buccolingual	5.46 (3.58)	5.37 (2.91)	0.685
Enamel mid-facial	1.22 (0.25)	1.22 (0.36)	0.603
Dentin mid-facial	1.57 (1.40)	1.50 (1.12)	0.538

* Mann–Whitney U-test is not significant.

**Table 7:** Correlation between tooth structure thickness and color values using Vita 3D-Master (VM) and Vita Easy Shade (VE).

Thickness	Overall						
		*L*	Chroma	H Degree	*a*	*b*	*WID*
Enamel-to-enamel buccolingual	VM	−0.216 *	0.315 *	−0.267 *	0.367 *	0.308 *	−0.385 *
	VE	−0.414 *	0.418 *	−0.512 *	0.521 *	0.417 *	−0.486 *
Enamel mid-facial	VM	−0.024	0.211 *	0.000	0.157	0.211 *	−0.228 *
	VE	−0.270 *	0.292 *	−0.335 *	0.339 *	0.292 *	−0.308 *
Dentin mid-facial	VM	−0.255 *	0.258 *	−0.298 *	0.351 *	0.252 *	−0.340 *
	VE	−3.90 *	0.374 *	−0.485 *	0.486 *	0.372 *	−0.453 *

* Spearman’s rho is significant at *p* < 0.05.

## Data Availability

The data will be available upon reasonable request.
